# Progress of Imaging in Diabetic Retinopathy—From the Past to the Present

**DOI:** 10.3390/diagnostics12071684

**Published:** 2022-07-11

**Authors:** Shintaro Horie, Kyoko Ohno-Matsui

**Affiliations:** 1 Department of Advanced Ophthalmic Imaging, Tokyo Medical and Dental University, Tokyo 113-8519, Japan; shinoph@tmd.ac.jp; 2 Department of Ophthalmology and Visual Science, Tokyo Medical and Dental University, Tokyo 113-8519, Japan

**Keywords:** diabetic retinopathy, retinal imaging, optical coherence tomography, scanning laser ophthalmoscopy, wide-field retinal imaging

## Abstract

Advancement of imaging technology in retinal diseases provides us more precise understanding and new insights into the diseases’ pathologies. Diabetic retinopathy (DR) is one of the leading causes of sight-threatening retinal diseases worldwide. Colour fundus photography and fluorescein angiography have long been golden standard methods in detecting retinal vascular pathology in this disease. One of the major advancements is macular observation given by optical coherence tomography (OCT). OCT dramatically improves the diagnostic quality in macular edema in DR. The technology of OCT is also applied to angiography (OCT angiograph: OCTA), which enables retinal vascular imaging without venous dye injection. Similar to OCTA, in terms of their low invasiveness, single blue color SLO image could be an alternative method in detecting non-perfused areas. Conventional optical photography has been gradually replaced to scanning laser ophthalmoscopy (SLO), which also make it possible to produce spectacular ultra-widefield (UWF) images. Since retinal vascular changes of DR are found in the whole retina up to periphery, it would be one of the best targets in UWF imaging. Additionally, evolvement of artificial intelligence (AI) has been applied to automated diagnosis of DR, and AI-based DR management is one of the major topics in this field. This review is trying to look back on the progress of imaging of DR comprehensively from the past to the present.

## 1. Introduction

Evolution in retinal imaging almost equally mean progress in the management of retinal diseases. Conventional optical photography has been a standard method for more than half a century. Since the early 1970s, standardized fundus photography in diabetic retinopathy (DR) has been evaluated [1,2]. Grading of DR from color fundus photography was studied in a Diabetic Retinopathy Study (DRS) group or Early Treatment Diabetic Retinopathy Study (ETDRS) group following the Airlie House classification of DR [3,4]. In the early days, color fundus photography was taken by film camera, and film has been gradually replaced by digital cameras, along with digital angiography with a scanning laser ophthalmoscope (SLO) [5]. More recently, color fundus imagery obtained by SLO has been also popular especially in obtaining wide-field images. Regardless of each type of optical technology or the field of view covered, color fundus imagery can show changes originated from lesions in any stages of DR, such as fundus examination by biomicroscope.

Fluorescein angiography (FA) is another vital examination in DR and retinal vascular diseases [6]. It has been a gold standard method in detecting various vessel changes found in DR, especially in finding retinal microaneurysms, non-perfusion areas, intraretinal microvascular abnormalities (IRMA), or retinal neovascularization with dye leakage [7,8,9]. Intravenously injected fluorescein sodium is excited by blue wavelength flashlight through an exciter filter, and the excited reflection is detected by either film or a digital system through the barrier filter. Since systemic administration of fluorescein iokljdye has potential risk of allergy or anaphylaxis in the most severe cases, the indication of the examination is limited [10,11].

Following the introduction of digitalization into the fundus camera, film-based printed photography has become digital image data. Stereoscopic photography has been less popular together with this, partly because optical coherence tomography (OCT) was introduced [12,13]. However, it is apparently useful in managing large numbers of clinical data with digitalized ones, and obtaining montage fundus photographs with digital images has been much easier than with printed photography. Digital images are also a technical basis of trial in automated diagnosis, which has been investigated since the early 2000s [14] and is applied to more recent evolution with artificial intelligence [15,16,17,18].

Subsequent evolution of the fundus camera was the introduction of the scanning laser ophthalmoscope (SLO). Scanning fundus by laser yields high-resolution images of retina, and ultra-widefield (UWF) imaging, even through small pupils, was dramatically launched with Optos (Optos plc) [19,20]. Retinal images of more than 100 degrees field of view are termed as UWF images, and DR with peripheral retinal lesions is one of the best indications of UWF imaging. In addition to laser with two or three colors (red, green, with or without blue), light-emitting diode (LED), is also used for light sources in scanning ophthalmoscopy [21].

Optical coherence tomography (OCT) is one of the most remarkable breakthroughs in retinal imaging and has greatly contributed to assessment of DR [22]. In eyes with diabetic macular edema (DME), OCT provides a large amount of information of the inner retinal structure to us. Today, OCT is an essential tool in the clinical management of DME. In addition to conventional tomographic images of the macula or retina, retinal vascular images such as FA without dye injection are provided with the more recent technology of OCT angiography [22,23,24,25,26]. The latest development in OCT angiography presents us widefield vascular images of retina, from which we can find non-perfused areas (NPAs) or neovascularization (NV) clearly.

## 2. Evolutions of Contemporary Modalities in Imaging of DR

### 2.1. Color Fundus Photography

History of fundus images obtained by fundus cameras dates to late 19th century, and modern fundus photographs were reported by Friedrich Dimmer in the early 20th century following the introduction of a commercial device from Carl Zeiss [27]. The first fundus atlas was published in 1927 by Dimmer’s associate Pilate, which included stereoscopic images by this time [28]. Color film and electronic flash were cooperated into photography in the pre- and post-war era. After 1955, a commercially easy affordable fundus camera was available worldwide, and fundus photography has become popular and fundamental data in ophthalmology. Later, ETDRS adopted stereoscopic seven-field photographs (30-degree color fundus fields) and examined in many clinical trials [4]. Stereopsis is obtained by utilizing a stereoscopic viewer. Stereopsis has been considered useful especially in detecting macular edema, traction retinal detachment, or distinguishing neovascularization (NV) on the retinal surface between intraretinal microvascular abnormalities (IRMA) in DR. However, in many clinics in the real world, non-stereoscopic photography of single or two-field (30- or 45-degree fields) is also a popular method in the management of DR. In terms of recording media for photography, film has been replaced by digital storage, which enables us to find images on a monitor on camera or transmit them to other computed devices, and stereoscopic photography has been less common in the era of digital data. Even though ETDRS seven-field standard photography may cover a majority of retinal lesions of DR, it is certainly not perfect in the entire vascular change of DR in periphery. Taking seven-field photographs is not sufficient in detecting peripheral lesion in DR, which is almost outside of seven-field standards [29,30,31]. To solve these problems, UWF retinal image obtained by single acting had been widely anticipated. Comparative studies between non-mydriatic or mydriatic UWF images and ETDRS seven-field standard images were investigated [32,33,34,35,36,37]. Automated DR diagnosis in fundus photography, one of the software with artificial intelligent was recently approved by FDA, adopts two-fields photographs [38], and such cost-effective screening of this disease is another hot topic.

### 2.2. Ultra-Widefield (UWF) Imaging and Confocal Scanning Laser Ophthalmoscope (SLO)

**Figure 1:**
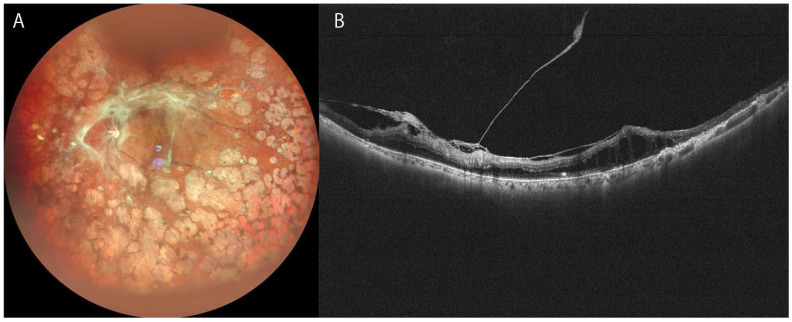
Ultra-widefield (UWF) fundus or OCT images of severe proliferative diabetic retinopathy of 74-year-old woman. (**A**): UWF image of Mirante, (**B**): UWF OCT of Canon S1.

**Figure 2:**
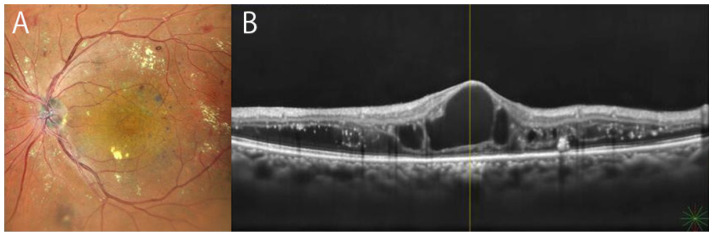
Posterior fundus and macular OCT images of diabetic macular edema of 62-year-old woman. (**A**): Synthesized color SLO image of Mirante, (**B**): Swept-source OCT of Mirante.

Since technology of SLO has been introduced to fundus photography in recent eras, a UWF retinal image can be obtained with a quick action. Not only is it easy to obtain broad images of the retina, UWF images help us understand characteristics of the fundus immediately. In previous eras, a montage of seven or more conventional field (30- or 45-degree) photographs has been accepted as whole or panoramic retinal fundus images for a long time. Confocal SLO for FA/indocyanine green angiography (ICGA) has been commercially available since early 1990s from Heidelberg Engineering, and their technology has been improved to supply multicolor WF photographic instruments with additional fundus autofluorescence (FAF) recently [39]. Concurrently, the concept of UWF photography covering maximum 200 degrees field of view has been developed by Optos Plc [19,20]. This technology is a milestone achievement in the history of fundus imaging, as 200-degree fields of view cover more than approximately 80% of the fundus area comparing to that of 30% in seven-field standards, and the new era of WF imagery started in modern clinical scene. In addition, the importance of detecting peripheral lesions in DR has been paid more attention, and comparative studies between UWF and conventional seven-field photographs were reported [32,33,34]. Following the breakthrough of UWF imaging by Optos, other ophthalmic industries launched WF/UWF technology. Clarus (133 degree) by Carl Zeiss [21] and Mirante (163 degree) by NIDEK [40], (Figure 1 and Figure 2), other devices for UWF imaging with various imaging modalities, are now commercially available (Table 1). Multicolor laser or light-emitting diode (LED) are adopted in the cameras, and they scan fundus thoroughly in order to obtain an entire image without mydriasis and avoiding stressful glaring of examinee. The characteristics of three currently available UWF cameras are summarized in Table 1. Ultra-wide field imaging has been applied to FA, and DR is one of the best indications of UWF-FA since DR is potentially involved not only in posterior fundus but in the entire area up to periphery. Retinal non-perfusion area and ischemia outside of ETDRS seven-fields has now been evaluated more accurately; this gives us new insight and tools in the management of DR.

### 2.3. Optical Coherence Tomography (OCT) and OCT Angiography (OCTA)

Development of OCT is one of the real breakthroughs in retinal imaging. Different from color fundus photography, it presents us internal information of the retina, choroid, or even posterior vitreous [22]. From the 1990s, application of OCT technology in ocular imaging was reported [12]. The first commercial OCT instrument was introduced by Carl Zeiss Inc., (Dresden, Germany). in 1996. The basal technology of first-generation OCT is time domain technology [41]. Subsequently, spectral domain (high-definition or Fourier domain) and swept-source technology developed into a more recent model with higher resolution, and wider or deeper range OCT images have been available [42,43,44,45]. This new imaging is useful especially in diagnosis of abnormalities in vitreoretinal interfaces or macular edema in DR [22] and is positioned as a first line of assessment for DME. Structural information both inside and outside of the retina has been visible in more detail along with the development of OCT technology. Recent swept-source OCT can show 10 layers of histological sections of the retina in high resolution. Exudative components derived from breakdown of the inner and outer retinal barrier are identified in exact locations as outer plexiform layer, inner nuclear layer, or subretinal space. Additionally, numerous critical pathological observations related to DME and prognosis has been found by this advancement of imaging. Furthermore, choroid has been more easily observable by introducing enhanced-depth imaging technology to OCT. Another trend of significant development in this imaging technology is the application of vascular imaging in the retina and choroid. This new modality is called as OCTA [23,24,46], in which detection of flow of erythrocyte in vessels is core technology. One of the notable advantages of OCTA is its three-dimensional ability to detect vascular flow in the retina, and that is distinct character by FA. We can distinguish capillary plexus in the different depth of layer by OCTA technology, which is impossible by FA. This technology enables us to identify more precise locations of vascular abnormalities, such as enlargement of the foveal avascular zone or microaneurysm of superficial or deep capillary plexus.

Recent swept source OCTA (OCT-S1, Canon) covers not only the macular area but also a wider field (21 × 23 mm) up to periphery [47], Figure 1.

### 2.4. Adaptive Optics

Technology of adaptive optics (AO) has been applied to retinal imaging in DR [48]. The AO-SLO camera enables us to observe retinal structure at the cellular level or the dynamics of the cell flow of the inner retina. High resolution AO-SLO imaging showed in vivo observation of micro-changes of capillary such as occlusion, recanalization, dilatation, resolution of local retinal hemorrhage, capillary hairpin formation, capillary bend formation, or microaneurysm formation in the macula area of DR patients [49]. This technology is especially of scientific value in retina research, obtaining a retinal image in extreme high magnification in vivo. RTX1 AO retinal camera provided by Imagine Eyes (Orsay, France) is the commercial instrument of this technology.

## 3. General Characteristics of Imaging in DR

### 3.1. Mild Non Proliferative Diabetic Retinopathy (NPDR)

According to the standard classification of AAO (2017), retinal microaneurysms (MAs) or moderate retinal hemorrhage are major retinal findings in mild to moderate diabetic retinopathy [50]. In fundus color photography, a red dot smaller than 125 microns in diameter with sharp margins in color fundus or FA is defined as MA [51] and is distinguished from hemorrhage. Fundus with MAs only indicates mild DR, and the diabetic eye with MAs plus hemorrhages, etc. is classified as more than moderate DR. FA is the most sensitive method in detecting retinal MAs and can distinguish them from retinal hemorrhages. Some of MAs are also detected by OCT as hyper-reflections with hyporeflective space inside. OCT angiography is superior to OCT section in detecting Mas, while OCTA cannot detect MAs without or slow flow below the threshold. Although FA is a more sensitive method in finding MAs, OCTA can distinguish whether it is existing in a superficial capillary plexus or a deep capillary plexus, which is difficult in FA [22,23,52]. Furthermore, OCTA can detect pre-clinical foveal microvascular change in DR [53].

### 3.2. Moderate to Severe NPDR

Severe NPDR is defined as the eye with severe intraretinal hemorrhage and MAs, venous beading in more than two quadrants, or any intraretinal microvascular abnormalities (IRMAs). Moderate NPDR is less than severe NPDR but more than the eye with MAs only in recent AAO classification [50]. NPDR includes most of the DR findings except retinal neovasculatization or vitreous hemorrhage and is based on seven standard fields of color photographs but FA. A retinal hemorrhage is found relatively easily in color photography but is difficult to find in FA. In OCT images, it presents as a moderate reflection and is not clearly defined. Hard exudates are also typical findings in NPDR, showing yellowish distinct appearance in color photographs as well as in biomicroscopes. The presence of hard exudates indicates chronic leakage from MAs or retinal vessels. In OCT, hard exudates are detected as hyper-reflection in various sizes, and look like tiny hyper-reflective foci, which is also possibly a precursor of hard exudates [54]. Following early changes of microcapillary structure, i.e., loss of pericytes, breaks of tight junction of capillary endothelium, or formations of Mas, loss of vascular unit of capillary networks connected arterioles or venules progress [55,56], also with disruption of neural networks [57], retinal area with loss of blood flow with obstructing vessels is termed as retinal nonperfused areas (NPAs), and it leads to tissue ischemia [55,56,57]. The ischemia also causes the release of vascular endothelial growth factor (VEGF) from retinal cells, and the cytokine induces the progression of stage from NPDR to proliferative DR (PDR). Treating identified NPAs by photocoagulation can possibly inhibit the development of neovascularization and worsening from NPDR to PDR [58,59,60]. FA is an outstanding method in detecting many DR findings in this stage, as it directly captures vascular networks and dynamics. FA has been the most common method in detecting NPAs in the clinical scene, and WF-FA is more useful in detecting the NPAs in the peripheral retina [61,62,63,64,65,66]. However, one of major problems is FA requires an intravenous injection of fluorescein sodium, and serious anaphylaxis cannot be perfectly prevented [10,11]. OCTA is a novel hopeful method for overcoming such problems of FA. OCTA detects and shows us high-resolution retinal blood flow as three-dimensional vessel structures [23,24,46], although it cannot show dynamics of blood flow and leakage from vessels. The advent of widefield OCTA has allowed clinicians to examine areas similar to ETDRS standard seven-fields avoiding serious side effects in examinee [66,67].

### 3.3. Proliferative Diabetic Retinopathy (PDR)

PDR is defined as the eye with any retinal neovascularization (NV). DR with vitreous hemorrhage is also included this stage because of highly supposed existence of retinal NVs of DR [3,51]. While color photography or biomicroscopes are useful in detecting NVs and basic methods of fundus examination, retinal NV is not always easy to find by these examinations. Regarding PDR, FA is one of the most sensitive methods in detecting NV, as it presents massive fluorescein dye leakage. Since NV is aberrant immature blood vessels lacking normal tight junctions, and invading and distributing in vitreous, fluorescein dye easily leaks from NV to vitreous and presents massive hyperfluorescence in the FA image. As mentioned above, disadvantage of FA is an invasive technique, and it cannot be performed on all DR patients [10,11]. OCTA is a novel alternative method for the angiography without need of dye injection. Although dye leakage from NV is not able to be shown in this method, pathological vascular networks can be more clearly defined without disturbance. Recent WF swept source OCTA can detect NVs on periphery, and this is a promising advancement of retinal imaging in this field [66,67,68,69]. However, obtaining a clear OCTA image of the periphery by conventional OCT devices is difficult especially in eyes with opacities in the media. In another point of view, direct observation of NV during vitrectomy with or without OCT combined in surgical microscope is also possible [70].

### 3.4. Diabetic Macular Edema (DME)

DME is one of the major complications as a sight threatening pathophysiology in DR and is characterized as intra-subretinal fluid with disorganization of macula. Stereoscopic photography of macular area has been one of the standard methods in detecting DME and included in ETDRS seven-field [52]. FA has been another gold standard method in diagnosing DME, since fluorescein dye pools in intraretinal cystoid space typically in Henle’s fiber layer or staining macular area with dye leakage from capillaries and vessels. In addition to these classical methods, OCT is a true milestone in the revolution of macular imaging [13]. Cystoid spaces, intraretinal fluid, or subretinal fluid are now easily visible in vivo by this method (Figure 2). Association of macular non-perfusion assessed by FA and central subfield thickness measured with OCT was examined [71]. OCT has elucidated numerous changes and understanding of this pathology in DME, such disorganization of retinal inner layers (DRIL) [72,73], hyper-reflective foci [54], or that disruption of Elipsoid zone or ELM indicates macular dysfunction [74,75,76]. The application of each imaging modality to any stages of DR in clinical settings is reviewed and summarized in Table 2. OCTA of the macular area is also clinically valuable for DME. Enlargement of the foveal avascular zone found in OCTA was reported in DME [77]. The association of chronic macular cysts and capillary non-perfusion in DME was investigated [78]. It was also reported that several biomarkers in OCTA images was improved following anti-VEGF injection [79].

## 4. Recent Topics in Imaging in DR

### 4.1. UWF Blue SLO Image in Detection of Retinal Ischemia

**Figure 3:**
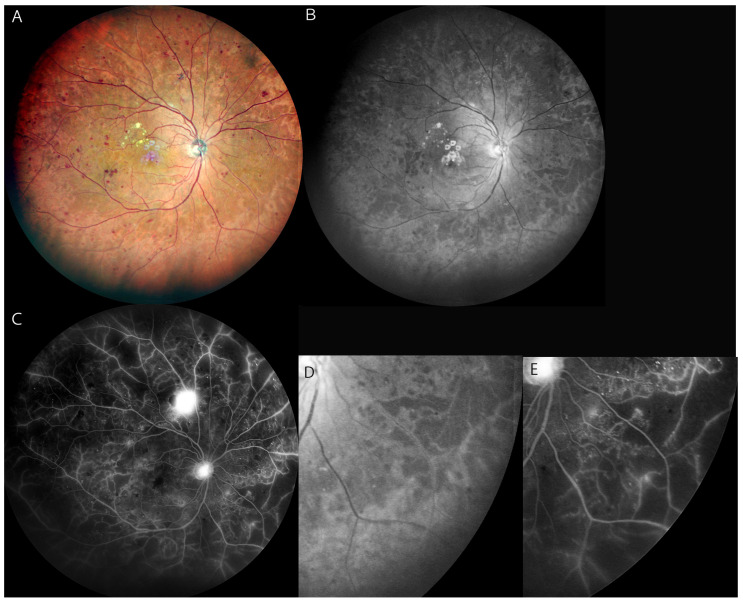
(**A**): Multicolor widefield SLO image of the right fundus of a 65-year-old man with PDR. (**B**): Blue SLO image shows a hyporeflective area in the mid-periphery to periphery of the fundus, indicating non-perfused areas. (**C**): Widefield FA image shows widespread NPAs extensively with dye leakage from new vessels. (**D**): Magnified image of image (**B**) shows hyporeflective areas in the lower temporal quadrant. (**E**): Magnified FA image of image (**C**) shows NPAs in the same quadrant of image (**D**). All figures above are Mirante (NIDEK, Japan) images.

The advantages of widefield image compared to conventional devices are not only its ease in obtaining images of wide areas of the fundus, but it also allows the acquisition of seamless images avoiding the absence of some areas of the far periphery. This is especially useful to find the retinal non-perfused areas in eyes with DR as much as possible. At present, UWF and multicolor SLO imaging can be performed by the Optos (Optos plc, UK), Mirante (NIDEK, Japan) and Clarus (Carl Zeiss, Germany) (Table 2). The Mirante device is a multimodal imaging ophthalmic instrument whose image covers a field of view of 163 degrees, narrower than 200 degrees field of view of Optos. The color images are obtained by three colors (red, green, and blue), while Optos is constituted with red and green colors. The additional blue light renders interesting character to the image of Mirante [40]. In the multicolor SLO images, each colors shows the lesion differently because of the differential absorption of each wavelength. There was a previous study reporting hyporeflectons in the red-free SLO highly corresponded with the NPAs in eyes with RVO and DR [80]. Since DR lesions are found throughout the retina including peripheral lesions, WF imaging is especially useful in finding NPAs. In our study, the blue WF-SLO image taken by Mirante showed fundus hyporeflective areas in the eyes with more than moderate to severe NPDR. Among the total 92 eyes, 73 eyes with more than moderate/severe NPDR had hyporeflective areas in the blue SLO images (79.3%). Additionally, substantial concordance between the hyporeflective areas of blue SLO and the NPAs of FA was confirmed (Cohen’s Kappa: 0.675). The location of the hyporeflective areas identified in the blue SLO images were found within the location of the NPAs of FA images in more than moderate to severe NPDR (Figure 3). In the UWF-OCT analysis, retinal thinning and disorganization were observed in hyporeflections, and we speculated it is one of the possible reasons why hyporeflections in blue SLO were found in ischemic retinas. The blue wavelength may easily penetrate the ischemic retinal layers with structural disorganization. According to this study, Mirante has practical value in the management of the eyes with referable DR because it sufficiently covers fundus fields with essential retinal information [40].

### 4.2. UWF-FA and Quantification of Retinal Non-Perfused Areas

Retinal pathology in DR is not only found in up to mid-peripheral retina but also in far periphery. Peripheral lesions identified in UWF image (Optos), also outside of ETDRS seven-field, may increase severity scale of DR [30]. Recently, DR lesions with a greater extent of outside vs. inside ETDRS standard seven-field is named as predominantly peripheral lesions (PPLs) [31], and the association with DR progression or severity is reported [81]. Current progress of UWF technology to FA enables us to find peripheral NPAs more easily and with high precision. Since FA shows us retinal NPAs as defection of capillary network, quantification of NPAs in UWF FA image of Optos with the combined software analysis is possible. One of the comprehensive analyses reported that 70% of NPAs are located outside the posterior pole, and increased NPA is associated with PPL found in UWF-FA [65]. UWF-FA analyzed to assess retinal leakage, ischemia, or count of Mas was also associated with DR stage [63]. The relation of quantified NPAs to DME [61,62], prognostic value [64], or the effect of anti-VEGF treatment on NPA [82] were reported. Additionally, fractal dimension (FD) analysis, which is a novel technique of quantification, has been applied to quantification of retinal vasculature [83,84]. Retinal vascular bed area (RVB) by FD analysis was examined in relation to severity of macular edema [85], NPAs [86], or DR stages [87,88].

### 4.3. WF-OCTA

OCT angiography (OCTA) is one of the major breakthroughs in retinal imaging. Commercial availability of this instrument started from 2014 by Optovue Inc (UK), followed by many manufacturers currently. Since this technology is detecting red blood cell flow of retinal perfusion but capillary or vessels walls themselves, vessels without or with quite low perfusion were not depicted. Compared to FA, the predominant characteristics of OCTA imagery are high-resolution capillary networks with different layers through chorioretinal depths. However, OCTA cannot detect leakage from vessels and dynamics of blood flow, like they are easily shown in FA. Recent models of this new modality such as Canon S1 OCTA covers maximum 23 mm × 20 mm area [47], and around 12 mm square area are covers in widely used instruments as PLEX Elite (Zeiss) [25,26,69]. The development of capturing broader areas in a single taking action substitutes panoramic montage OCTA image and has great potential in evaluating NPAs in future. Comparing the detection rate of any DR abnormality among OCTA and ETDRS seven-field color fundus photography was studied, and the rate of IRMA was especially higher in WF-OCTA [25]. WF-OCTA could detect NPA [66] or NV [66,68] rate similar to UWF-FA at a high rate.

However, since leakage from vessels cannot be shown in this way, retinal neovascularization should be carefully inspected compared to FA. Additionally, it is still not easy to capture fine images constantly because it is easy to be influenced by eye movement or media opacity. Therefore, OCTA is developing technology so far, but has great potential in substituting FA and may be a sufficient method for detection of NPA, NV, or IRMA in DR [89].

### 4.4. Automated Diagnosis in DR

Given the high prevalence and social importance of screening DR, it has been one of the best and popular target diseases of automated diagnosis. Automated retinal image analysis software has potential to substitute conventional photo-based screening by ophthalmologist or physicians [90]. IDx-DR (Digital Diagnostics) was the first artificial intelligence diagnostic software for DR screening approved by FDA and combined with automated non-mydriasis fundus camera of Topcon. Following the approval from FDA, it has been introduced in several countries and areas. This system diagnosis with two conventional 45-degree photographs of macular-centered and disc-centered and was reported to offer sufficient quality of detecting referable DR compared to human grading [16]. Many studies also reported high rate of sensitivity and specificity of autonomous-AI diagnosis system in diagnosing any stage of DR [38,90,91,92,93,94,95]. Diagnostic sensitivity of the algorithm (2iRetinex) for DR to sight threatening vision was examined, and the sensitivity is nearly identical to diagnosis by physician [92]. However, the AI-based diagnosis system is based on two-dimensional photography with limited retinal areas than seven-fields standard or wide-field image. Lack of stereoscopic observation possibly influences the detection of macular edema or fibrovascular change of PDR. Additionally, recent UWF imaging could detect peripheral dominant lesions of DR. Comparing automated diagnosis with a narrower field of image to UWF imaging diagnosed by retina specialist should be discussed in the future.

## 5. Discussion

Since the history of retinal imaging in the eyes with DR initiated from fundus film-based photography, the method has been evolving to depict more magnified or wider retinal images with less invasiveness to the examinee. In addition to fundus photography, OCT is another new powerful technique of retinal imaging, which enables us to watch the live status of pathological changes of DR.

ETDRS stereoscopic seven-field standard photography has long been a golden standard in the diagnosis and evaluation of DR, especially in investigating studies. Clinically, the major sight-threatening pathological changes of DR are edema or retinal detachment in the macular area other than persistent vitreous hemorrhage. Stereoscopic evaluation of the macular area and fibrovascular proliferation lineated vascular arcade or optic disc are well-identified and covered by this classical method. However, obtaining stereoscopic photographs requires much more effort, and advantages of this method can be substituted by OCT or direct fundus examination by biomicroscope.

One of the trends in evolution of retinal imaging is capturing a wider area in single photograph [96]. Before introducing widefield imaging technology based on scanning light sources, a montage image had been frequently made with classical 30/45 degrees of field photographs. Recent technology with scanning with multiple colors of lasers or LED replaced such incomplete “widefield” retinal images as montage photographs are quite difficult to be made seamlessly. Moreover, the new technology is coupled with capturing images even without mydriasis. Optos (Optos plc, UK) is the pioneer of this kind of new generation of fundus photography and has been a synonym of UWF photography, as it covers the widest field of view (200 degrees). Later introduced UWF instruments are Mirante (NIDEK, Japan) and Clarus (Carl Zeiss, Germany), and these are characterized as three-color synthetic images, which aim to obtain realistic retinal images. Additionally, the image of single-component colors has its own characteristics and has great potential in diagnosis of retinal diseases [97,98,99,100,101]. The blue SLO image of Mirante can detect retinal non-perfused area without any dye injection and has potential to substitute FA [40]. The advantage and demand of widefield imaging in DR clinics is in no doubt, and it will be utilized more in practice soon.

Another trend in this field is given by the advancement of OCT technology. OCT of the new generation as swept-source instruments shows us the inside of the retina or even of choroid, like histological sections in high resolution in vivo. With this technology, we now can evaluate or predict macular function. The technology is now not only limited to the macular area but is also applied to ultra-widefield imaging [42,43,44,45,47]. Another advantage of OCT technology is its characteristic of relatively low invasiveness. OCT can replace the necessity of FA in evaluating macular disease such as DME, and widefield OCTA can be a great alternative to find retinal non-perfusions in DR.

In addition to these novel technologies, automated diagnosis by artificial intelligence is the most noteworthy topic in DR imaging, and many screening programs with or without telemedicine have been studied [102]. Smartphone-based automated diagnosing software was also reported [103] and has great potential in management of DR for the under-resourced African population [104]. In the future, it is prospected that technology of UWF fundus images, sectional OCT or OCTA will be continuously developed, and trial of auto-diagnosis by AI will be also combined to these modalities, thus more people will be diagnosed precisely with less effort worldwide.

## Figures and Tables

**Table 1:** Characteristics of ultra-widefield ophthalmoscopy.

Fundus Ophthalmoscopy/Camera	Optos (Optos Plc)	Mirante (NIDEK)	Clarus (Carl Zeiss)
Maximum field of view	200°	163°	133°
Without mydriasis	Yes	Yes	Yes
Light sources	Laser (red and green)	Laser (red, green, and blue)	LED (red, green, and blue)
Multimodality	FA, ICGA, FAF, OCT	FA, ICGA, FAF, Retromode, OCT, OCTA	FA, FAF
Features	Widest field	Mutlimodality	Color reality

LED: light emitting diode, FA: Fluorescein angiography, ICGA: Indocyanin green angiography, FAF: Fundus autofluorescence, OCT: optical coherence tomography, OCTA: OCT angiography.

**Table 2:** Clinical applications of imaging methods to any stages of diabetic retinopathy.

	Less than Mild NPDR	Moderate/Severe NPDR	PDR	DME
Biomicroscopy (contact or non-contact lens)	S	H	H	H
Color photography (1 or 2-field, 30/45°)	S	S	L	H
Color photography (7-field of 30°)	S	S	S	S
UWF color (>100°)	L	H	H	L
FA (30/45°)	N	S	H	H
UWF FA (>100°)	N	H	H	L
OCT	S	H	H	H
WF OCTA (12 mm × 12 mm)	N	H	H	L

NPDR: Non-proliferative diabetic retinopathy, PDR: Proliferative diabetic retinopathy, DME: Diabetic macular edema, FA: Fluorescein angiography, UWF: Ultra-widefield, OCT: optical coherence tomography, OCTA: OCT angiography, Applicability—N: No, L: Low, S: Substantial, H: High.

## Data Availability

Not applicable.
